# An infectious recombinant foot-and-mouth disease virus expressing a fluorescent marker protein

**DOI:** 10.1099/vir.0.052308-0

**Published:** 2013-07

**Authors:** Julian Seago, Nicholas Juleff, Katy Moffat, Stephen Berryman, John M. Christie, Bryan Charleston, Terry Jackson

**Affiliations:** 1 The Pirbright Institute, Woking, Surrey GU24 0NF, UK; 2 Institute of Molecular Cell and Systems Biology, College of Medical, Veterinary and Life Sciences, University of Glasgow, Glasgow G12 8QQ, UK

## Abstract

Foot-and-mouth disease virus (FMDV) is one of the most extensively studied animal pathogens because it remains a major threat to livestock economies worldwide. However, the dynamics of FMDV infection are still poorly understood. The application of reverse genetics provides the opportunity to generate molecular tools to further dissect the FMDV life cycle. Here, we have used reverse genetics to determine the capsid packaging limitations for a selected insertion site in the FMDV genome. We show that exogenous RNA up to a defined length can be stably introduced into the FMDV genome, whereas larger insertions are excised by recombination events. This led us to construct a recombinant FMDV expressing the fluorescent marker protein, termed iLOV. Characterization of infectious iLOV-FMDV showed the virus has a plaque morphology and rate of growth similar to the parental virus. In addition, we show that cells infected with iLOV-FMDV are easily differentiated by flow cytometry using the inherent fluorescence of iLOV and that cells infected with iLOV-FMDV can be monitored in real-time with fluorescence microscopy. iLOV-FMDV therefore offers a unique tool to characterize FMDV infection *in vitro*, and its applications for *in vivo* studies are discussed.

## Introduction

Foot-and-mouth disease virus (FMDV) is the aetiological agent of foot-and-mouth disease (FMD) of cloven-hoofed animals. FMDV is highly contagious, and outbreaks are a major threat to global food security due to their devastating economic effects. FMDV belongs to the genus *Aphthovirus* of the family *Picornaviridae* and has a positive-sense single-stranded RNA genome encapsidated within a non-enveloped icosahedral shell. An internal ribosome entry site facilitates translation of the FMDV genome, yielding a polyprotein that is subsequently processed to a number of intermediate products and 12 mature proteins: the non-structural auto-proteinase (L^pro^); the structural proteins VP4 (1A), VP2 (1B), VP3 (1C) and VP1 (1D) and the remaining non-structural proteins (nsp) 2A, 2B, 2C, 3A, 3B, 3C^pro^ and 3D^pol^ (Forss *et al.*, 1984; Rueckert, 1996). During translation of the FMDV polyprotein, an intra-ribosomal self-processing event occurs at the C terminus of 2A separating the region containing the capsid proteins (P1) from most of the non-structural proteins. 2A is subsequently cleaved from P1 at its N-terminal by the 3C protease, which is responsible for the majority of the remaining proteolytic cleavages in the FMDV polyprotein.

In 1898, FMDV was the first animal disease shown to be caused by a virus (Rott & Siddell, 1998). Over a century of active investigation has led to the elucidation of many aspects of the FMDV life cycle; however, critical knowledge about virus–host interactions is still lacking, including the complete identification of host cell types permissive to infection and the characterization of viral dissemination following known routes of natural infection. At present, identification of the host cell repertoire targeted by FMDV during infection relies on the use of specific antibodies that detect viral non-structural proteins in permeabilized cells, an invasive technique that does not facilitate the visualization of these nsp in real-time as an infection progresses. With this in mind, we decided to initiate the development of a recombinant FMDV expressing an easily detectable fluorescent marker that can be used to further characterize the FMDV life cycle.

The ability to successfully generate a recombinant infectious picornavirus that expresses a fluorescent marker protein is dependent on several factors, including the site of insertion targeted in the viral genome, the size and stability of the exogenous RNA that is inserted, the packaging limits imposed by the viral capsid and the expression and detection of the encoded fluorescent protein. In this study, we used a reverse genetics approach and the inherent processing events that occur either side of the 2A product within the FMDV polyprotein to produce recombinant FMDVs (rFMDVs) containing different exogenous RNA insertions. Furthermore, characterization of these rFMDVs provided insight into capsid packaging limitations for the selected insertion site, allowing us to construct, characterize and report for the first time an infectious fluorescent rFMDV.

## Results

### GFP-FMDV and *Renilla* luciferase-FMDV are non-viable and function as replicons

**Fig. 1.**
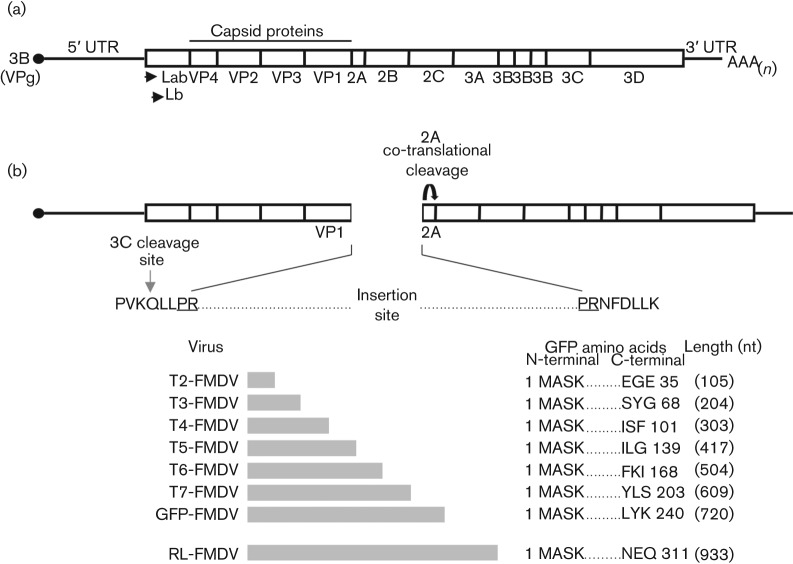
Cloning strategy utilized to construct FMDV infectious copy plasmids encoding the *Renilla* luciferase protein (RL) or different portions of GFP. (a) Schematic representation of the FMDV genome and encoded protein products. (b) Schematic representation of the FMDV genome site utilized for the insertion of the RL gene or different portions of the GFP gene. The retained 3C cleavage site at the C terminus of VP1 and the amino acids (underlined) encoded by the introduced *Avr*II restriction endonuclease site used for cloning the insertions are shown. The RL and different GFP insertions are depicted below, along with their respective nomenclature (T2- to T7-FMDV, GFP-FMDV, RL-FMDV), nt length (in parentheses) and encoded peptide.

In order to better study the FMDV life cycle, a reverse genetics approach was utilized to generate recombinant infectious copy viruses designed to express either the GFP of *Aequorea victoria* or the *Renilla* luciferase protein (RL) of *Renilla reniformis*, as an N-terminal fusion product of the non-structural FMDV 2A protein (Fig. 1). The genomic site utilized for insertion of the GFP or RL ORF was selected in order to retain the auto-catalytic cleavage of the 2A product at its C terminus. This cleavage facilitates the release of the P1 region from the nascent polyprotein. Similarly, the 3C protease cleavage site at the C-terminal of the VP1 capsid protein was retained to facilitate release of the N terminus of the 2A fusion protein (GFP-2A).

**Fig. 2.**
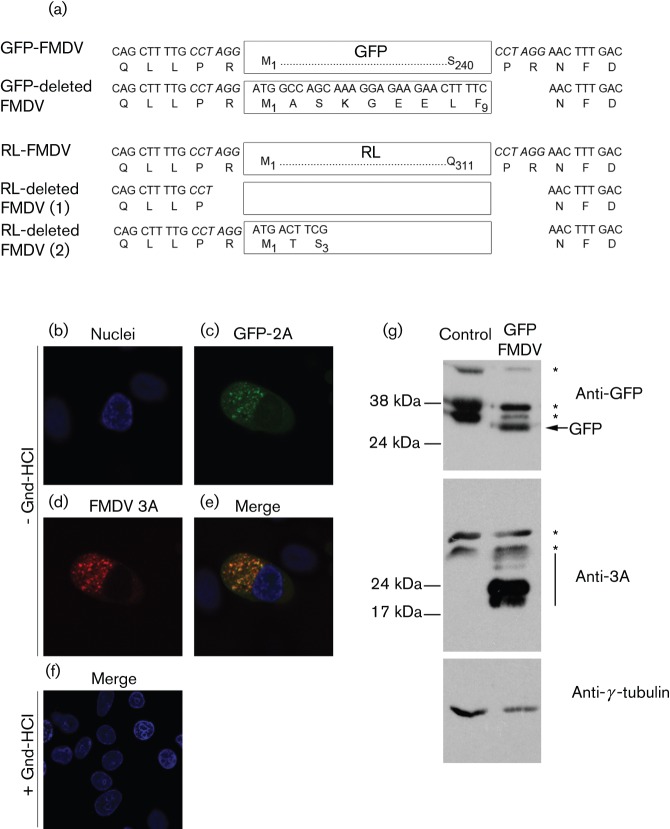
GFP and *Renilla* luciferase (RL) ORF insertions into the FMDV genome are deleted. (a) Sequence analysis of the single GFP-FMDV and two RL-FMDV deletion variants. The remaining amino acids of each insertion, as well as those flanking each deletion are shown [see GFP-deleted FMDV and RL-deleted FMDV (1) and (2)]. The amino acids flanking the undeleted insertions are also shown for reference (see GFP-FMDV and RL-FMDV). (b–f) BHK-21 cells transfected with GFP-FMDV and treated with the viral replication inhibitor guanidine hydrochloride (+ Gnd-HCl) or left untreated (− Gnd-HCl). GFP-FMDV replication was clearly visible in the untreated cells as judged by the expression of GFP (green, c) and the non-structural 3A protein (red, d). In comparison, no obvious replication was observed in the Gnd-HCl-treated cells (f). Nuclei are stained blue (DAPI). (g) Confirmation by Western blot analysis of GFP-FMDV replication in BHK-21 cells. Whole-cell lysates were analysed for GFP, the non-structural FMDV 3A protein (and 3A/B precursors, black bar) and γ-tubulin (loading control). Asterisks indicate non-specific bands.

With the intention of generating viral stocks, transcripts made from the GFP infectious clone were first electroporated into BHK-21 cells (passage 0 stock, P0). Whole-cell lysates prepared from the electroporated cells were then used to infect goat epithelium cells (P1) expressing the principal FMDV receptor, integrin αvβ6 (Jackson *et al.*, 2000). Three additional passages were performed in the goat epithelium cells, producing P2 to P4 stocks. During these consecutive passages, cytopathic effect (CPE) was only observed in goat epithelium cells infected with the P3 virus stock. In addition, we were unable to detect GFP expression by Western blot analysis and fluorescence microscopy (data not shown), in any of the virus stocks. Picornaviruses including FMDV are notorious for their recombination abilities; therefore, sequence analysis of the P4 virus stocks was carried out to check for the presence of an intact GFP ORF. As expected, the sequence data revealed a single virus population (at the consensus level) that had lost the inserted GFP ORF (Fig. 2a). Similarly, we were unable to generate infectious virus expressing RL when the encoding ORF was inserted into the same site of the FMDV genome. Once again, CPE was only observed in goat epithelium cells infected with the P3 virus stock. Sequence analysis of the P4 virus stock revealed two virus subpopulations (at the consensus level) that had lost most, if not all, of the inserted RL ORF (Fig. 2a). We therefore investigated the inability of the FMDV genome to retain the GFP insertion. At first, experiments were carried out to exclude the possibility of the FMDV genome being unable to competently replicate in the presence of the GFP sequence. To do this, BHK-21 cells were transfected with full-length RNA prepared from the GFP-FMDV infectious copy plasmid, and a subset was subsequently treated with guanidine-HCl, a potent inhibitor of FMDV replication (De Palma *et al.*, 2008; Rott & Siddell, 1998). Fig. 2 (b−e) shows that in untreated cells GFP-2A is clearly expressed following transfection; in comparison, no GFP-2A expression was observed following guanidine-HCl treatment (Fig. 2f). Viral replication was confirmed in the untreated cells by immunofluorescence labelling for the non-structural 3A protein. In addition, Western blot analysis further verified the expression of the GFP and 3A proteins (Fig. 2g).

### Determination of the packaging limitations for the targeted insertion site

**Fig. 3.**
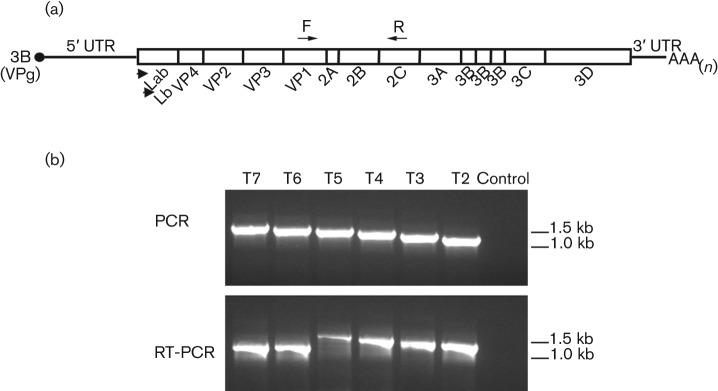
Determination of the packaging limitations for the targeted insertion site of the FMDV genome. (a) Schematic representation of the FMDV genome, showing the positions of the forward (F) and reverse (R) primers used to investigate the retention of different portions of the GFP gene. (b) Top: PCR controls performed using the T2- to T7-FMDV infectious copy plasmids as templates (see Fig. 1b). The PCR products show the expected amplification products derived from each of the FMDV genomes in the absence of deletions. Bottom: RT-PCR analysis of FMDV genomes (T2- to T7-FMDV) containing different portions of the GFP gene. FMDV genomes were prepared from goat epithelium cells infected with P0 virus stocks. T6- and T7-FMDV clearly show deletion of their respective insertions.

The ability to replicate its genome but not yield infectious virus suggested the GFP-FMDV was functioning as a replicon. We therefore decided to investigate whether the observed inability to generate infectious virus was a result of exceeding the packaging limitations imposed by the rigid FMDV capsid. Utilizing the same insertion site within the FMDV genome, a series of six infectious clones were constructed that contained increasingly larger portions of the GFP ORF (T2-FMDV (100 nt), T3-FMDV (200 nt), T4-FMDV (300 nt), T5-FMDV (400 nt), T6-FMDV (500 nt) and T7-FMDV (600 nt) (Fig. 1b). In contrast to the full-length GFP-FMDV, all six truncated GFP-FMDVs caused CPE in goat epithelium cells infected with the respective P0 viral stock, indicating the presence of infectious virus. To confirm the stability of each insertion, reverse transcriptase PCR (RT-PCR) was performed on these P1 virus stocks. Fig. 3(b) clearly shows that GFP portions ≤300 nt in length were retained by their respective viruses (T2-FMDV, T3-FMDV and T4-FMDV), whereas portions ≥500 nt were lost (T6-FMDV and T7-FMDV). Interestingly, RT-PCR carried out on T5-FMDV indicated the presence of a mixed virus population consisting of FMDV that had either retained or lost its 400 nt insert. Sequence analysis (data not shown) confirmed these results, indicating the maximum size of RNA that could be inserted into the targeted region of the FMDV genome, with regard to retaining the insertion over two passages, was 300–400 nt.

### Generation and characterization of an alternative fluorescent FMDV

The ORFs of full-length GFP (∼700 nt) and RL (∼800 nt) both exceed our proposed packaging limit (∼400 nt) for the selected insertion site. Recently, Chapman and colleagues engineered a smaller alternative fluorescent protein to GFP termed iLOV (Chapman *et al.*, 2008). Derived from a domain of phototropin, a plant blue light receptor, iLOV has an ORF of ∼300 nt and encodes a protein of ∼10 kDa. We hypothesized that inclusion of the iLOV ORF would fit the established constraints of the FMDV capsid and decided to explore the possibility of using the iLOV protein as an alternative fluorescent medium with which to study the FMDV life cycle. A codon-optimized version for mammalian cell expression of the DNA sequence encoding iLOV was inserted into the targeted site of the genome to create an iLOV-FMDV infectious copy virus.

**Fig. 4.**
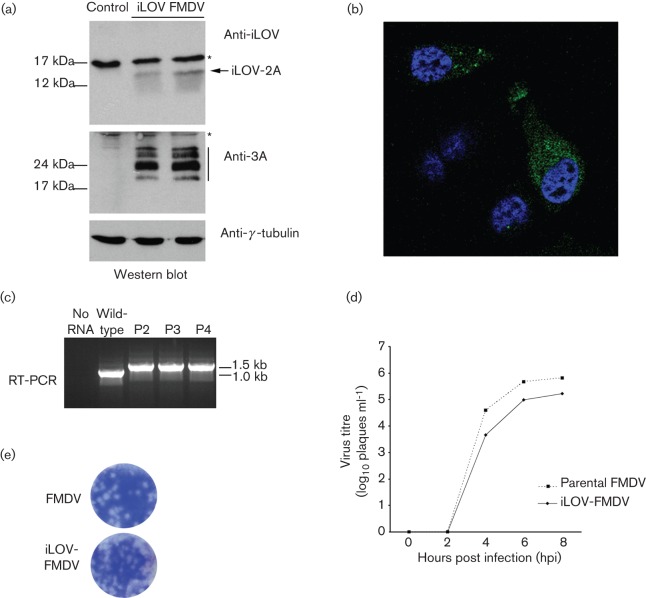
iLOV-FMDV is viable and stable. (a) Western blot analysis of whole-cell lysates prepared from goat epithelium cells infected with iLOV-FMDV. Samples were probed for the presence of iLOV, the non-structural FMDV 3A protein (and 3A-B precursors, black bar) and γ-tubulin (loading control). Asterisks indicate non-specific bands. (b) Fluorescence microscopy of goat epithelium cells infected with iLOV-FMDV. Nuclei are stained blue (DAPI). (c) RT-PCR analysis of iLOV-FMDV genomes prepared from P2, P3 and P4 virus stocks. Genome derived from the parental wild-type virus was also analysed as a control. (d) Growth analysis comparison of parental wild-type FMDV and iLOV-FMDV. Goat epithelium cells were infected with the respective virus (0.1 m.o.i.) and samples analysed at 0, 2, 4, 6 and 8 h post-infection. Similar results were obtained from three individual experiments. (e) Plaque morphology of goat epithelium cells infected with parental wild-type FMDV or iLOV-FMDV.

Viral transcripts made from the iLOV-FMDV infectious clone were electroporated into BHK-21 cells, which were used to prepare P0 virus stock that was used to infect goat epithelium cells. Indeed, P0 stocks of iLOV-FMDV were able to cause CPE in goat epithelial cells. Western blot analysis using rabbit sera recognizing the iLOV protein showed expression of a product of the predicted molecular size (Fig. 4a), confirming expression of the encoded N-terminal iLOV-2A fusion product. In order to confirm viral replication, expression of the non-structural 3A protein was also verified by Western blot (Fig. 4a). In addition, fluorescence microscopy was performed to corroborate the expression of the iLOV-2A protein in goat epithelial cells infected with P1 virus stock (Fig. 4b).

Next, we investigated the genetic stability of iLOV-FMDV in cell culture. Following the initial passage of iLOV-FMDV in electroporated BHK-21, virus was serially passaged four more times in goat epithelium cells to yield P0 to P4 virus stocks. Analysis of the P2, P3 and P4 stocks by RT-PCR revealed that exogenous iLOV DNA was stably retained within the FMDV genome over five passages (P0 to P4) (Fig. 4c). Sequence analysis of the entire capsid region confirmed these results and revealed no mutations (data not shown). Although our experiments clearly showed FMDV tolerated the iLOV RNA in terms of both its packaging capacity and retention, we reasoned that the insertion of an additional ∼300 nt into the genome would decrease the temporal efficiency of replication and possibly that of encapsidation. We therefore performed growth comparisons between the parental virus and iLOV-FMDV (Fig. 4d). As expected, the single-step growth kinetics exhibited by iLOV-FMDV showed a slightly reduced rate of growth in goat epithelium cells compared to the parental virus. To corroborate these results, we infected confluent monolayers of goat epithelium cells with either the parental-FMDV or iLOV-FMDV and compared their plaque morphologies (Fig. 4e); plaques exhibited similar morphologies, indicating similar rates of replication.

**Fig. 5.**
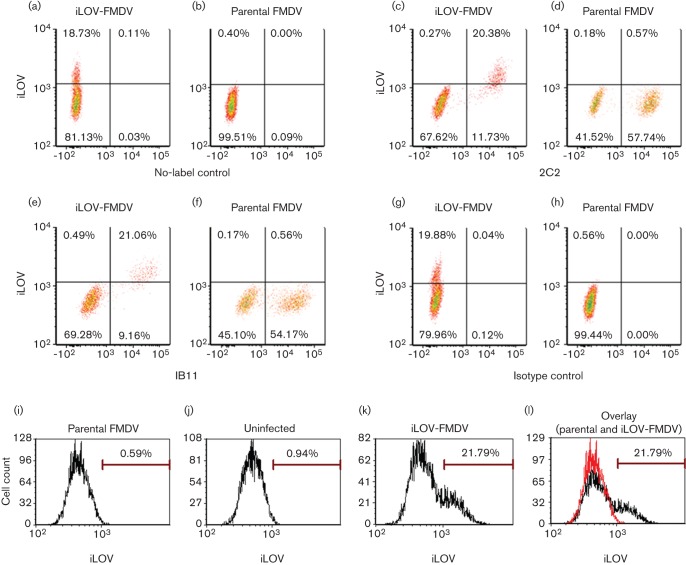
Detection of iLOV-FMDV-infected cells by flow cytometry. Goat epithelium cells were either mock infected or infected with iLOV-FMDV or parental FMDV at an m.o.i. of 2. After 4 h, cells were analysed by flow cytometry. (a–h) Density plots for iLOV-FMDV-infected or parental-FMDV-infected cells. (a, b) Infected and unlabelled cells analysed for iLOV expression. (c, d) Infected cells analysed for iLOV and FMDV non-structural protein 3A expression as detected with mAb 2C2. (e, f) Infected cells analysed for iLOV and FMDV capsid expression as detected with mAb IB11. (g, h) Infected cells analysed for iLOV expression and labelled with isotype control mAbs TRT1 and TRT3. (i–l) Histograms displayed for unlabelled cells infected with parental FMDV (i), mock infected (j), infected with iLOV-FMDV (k) and overlay of parental-FMDV-infected (red) and ILOV-FMDV-infected (black) cells (l).

**Fig. 6.**
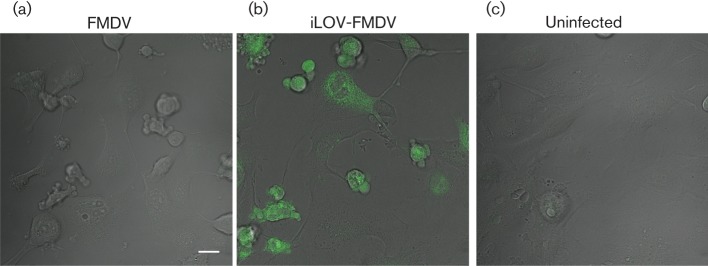
Detection of iLOV-FMDV-infected cells by live-cell imaging. (a–c) Still images from live-cell imaging experiments during which goat epithelium cells were infected at an m.o.i. of 2 with either parental-FMDV (a) or iLOV-FMDV (b) or mock infected (c). Cells imaged by differential interference contrast optics are shown. iLOV-expressing cells (green) are clearly visible in (b). Bar, 20 µm.

The above results show iLOV-FMDV replication is not drastically attenuated *in vitro*. The construction of a fluorescent FMDV offers a possible system to detect and purify infected cells prepared from *in vitro* and *in vivo* studies. With this in mind, we decided to explore applicable techniques by which iLOV-FMDV replication can be detected and quantified in cells, including flow cytometry and live-cell imaging. Fig. 5 shows that we were able to detect and quantify iLOV-FMDV-infected goat epithelium cells using flow cytometry; iLov expression was only detected in cells that were positive for both structural and non-structural proteins of FMDV. Lower percentages of iLOV-FMDV-infected cells were detected using anti-FMDV structural and non-structural antibodies compared with parental-FMDV-infected cells, which is consistent with a slightly reduced rate of growth. Live-cell imaging of iLOV-FMDV-infected goat epithelium cells (Fig. 6 and Video S1, available in JGV** Online) confirmed our initial fluorescence microscopy observations of fixed cells. iLOV-2A protein was clearly visible in infected goat cells, which exhibited the well characterized phenotype of rounding and cell death (CPE) in the later stages of infection.

## Discussion

The work herein investigates the packaging limitations of the FMDV capsid for a specific insertion site and explains why previous attempts to construct FMDV-encoding fluorescent proteins, such as GFP, failed. In addition, it provides further evidence that FMDV is able to remove insertions from its genome that exceed the packaging limits, possibly by fortuitous recombination events that maintain the integrity of the encoded viral proteins. Importantly, we describe for the first time the rescue of viable FMDV expressing a fluorescent protein, iLOV. Although not formally established, it is likely that rescue of the iLOV-expressing virus was successful because the coding region of this protein is within the limits tolerated by the virus. The iLOV insertion was genetically stable over five passages through cell culture. iLOV fluorescence was readily detected by confocal microscopy and flow cytometry, making it a very useful tool to study FMDV pathogenesis.

We targeted the junction between P1 and 2A as an insertion site for exogenous sequences to produce N-terminal fusion products of the 2A non-structural protein that were released from the FMDV polyprotein via the natural processing of 2A and the 3C^pro^ cleavage site at the C terminus of the VP1 structural protein. Similar reverse genetics approaches have been used to investigate the potential use of poliovirus vectors as vaccines for the expression of foreign antigens. A number of these viruses expressed their antigens either (i) as N-terminal additions to the poliovirus polyprotein or (ii) as an intermediate product at the junction between precursors P1 and P2 (between the VP1 and 2A products). In both cases, additional 3C^pro^ cleavage sites facilitated the release of product (the antigen) from the polyprotein (Andino *et al.*, 1994; Mattion *et al.*, 1994, 1995; Mueller & Wimmer, 1998; Yim *et al.*, 1996). These cloning strategies are particularly applicable to the arrangement of picornaviral genomes. Unlike the poliovirus 2A product, which is a bona fide protease that cleaves its own amino terminus auto-catalytically, FMDV 2A is processed at its C terminus during translation of the polyprotein by an autonomous intra-ribosomal self-processing event.

Initial attempts to generate rFMDVs expressing GFP or RL were unsuccessful, and all infectious progeny viruses contained deletions of most, if not all, of their respective exogenous ORFs. Similar results have been observed in a study with poliovirus, in which the insertion of the GFP ORF severely impaired viral replication and was deleted in the course of serial passage in cell culture (Mueller & Wimmer, 1998). Unlike this study, although we observed the replication-dependent expression of GFP-2A in cells transfected with GFP-FMDV transcripts, we were unable to generate any GFP-expressing infectious viruses. These results show the insertion of the GFP ORF into the FMDV genome imposed replicon-like characteristics. Replicons bypass cell entry and capsid uncoating, enabling the analysis of interactions to focus solely on the early events of viral replication. The use of GFP-FMDV and RL-FMDV as replicons that encode the complete repertoire of viral proteins is currently being investigated.

The reasons for the discrepancy between poliovirus and FMDV with respect to the ability to generate infectious GFP-expressing virus may involve the different sites of the genome targeted for insertion, as well as differences in the capsid packaging limits of the two viruses and the lengths of their genomes. Compared to the poliovirus genome (∼7.5 kb), the FMDV genome is larger (∼8.2 kb), and the difference is comparable in size to the GFP ORF (∼700 nt). Deletion variants that had lost their GFP or RL ORF insertion were only observed, as judged by CPE, after five consecutive passages. In addition, sequence analysis revealed the presence of only a few deletion variants; one in the case of GFP, and two for RL. This suggests that the generation of stable variants was a rare occurrence; however, the mechanism by which they were generated is unknown. All three of the deleted viruses exhibited almost precise deletion of their respective insert; however, it is likely that the packaging limits per se were not the only driving force for such complete excision. Mueller & Wimmer (1998) observed similar deletion events leading to the loss of foreign sequences that had been inserted into the poliovirus genome and proposed two models of illegitimate (non-homologous) recombination as possible mechanisms. Both models are based on the presence of short direct sequence repeats in the immediate vicinity of the deletion borders. In our study, short direct sequence repeats were present on either side of the sequences inserted into the FMDV genome in the form of *Avr*II restriction sites (CCTAGG), but sequence analysis of the deletion borders could not confirm a role for these sites in the recombination events. Numerous reports have detailed the ability of picornaviruses to generate extensive genetic variation, due to the unedited mis-incorporation of nts during RNA synthesis, recombination and other genomic rearrangements (Agol, 1997; Lukashev, 2010; Savolainen-Kopra & Blomqvist, 2010), and it is possible that more than one mechanism of loss was responsible for the generation of the stable variants. It is also possible that the codon usage bias in FMDV may influence the excision process (Zhou *et al.*, 2013).

In order to determine the size of exogenous RNA that could be stably inserted into the FMDV genome using the selected target site between P1 and 2A, we constructed a series of rFMDVs each containing an increasingly larger portion of the GFP ORF. Our experiments show that insertions up to 400 nt in length are more stable. These results are in agreement with poliovirus studies, which showed recombinant viruses based on polyprotein fusion or dicistronic expression are more stable if the foreign ORF is <400 nt (Lu *et al.*, 1995; Mattion *et al.*, 1994, 1995; Mueller & Wimmer, 1998; Yim *et al.*, 1996).

It is feasible to suggest that the constraints of the capsid prevented the GFP-FMDV genome from being packaged. Nevertheless, a number of factors other than the physical ability to package have been shown to influence the stability of foreign ORFs within the poliovirus genome, including replication competence, the presence of signal sequences or fortuitous RNA structures and the processing of introduced cleavage sites (Lu *et al.*, 1995; Lu & Wimmer, 1996; Mattion *et al.*, 1994, 1995; Yim *et al.*, 1996).

The β-barrel structure of GFP is essential to its function, inhibiting the production of smaller derivatives that could be more easily accommodated by the FMDV capsid (Cubitt *et al.*, 1995; Ormö *et al.*, 1996; Tsien, 1998; Yang *et al.*, 1996). We therefore decided to investigate the use of iLOV (Chapman *et al.*, 2008) as an alternative marker for FMDV replication. iLOV was created from the LOV2 domain (amino acids 387–496) of the phototropin 2 (phot2) plant blue light receptor of *Arabidopsis thaliana* by Chapman *et al.* (2008) using molecular evolution and tobacco mosaic virus (TMV)-based expression screening techniques. Chapman and co-workers also optimized iLOV codon usage for expression in mammalian cells, demonstrating that iLOV fluorescence can be detected in human embryonic kidney (HEK) cells (Chapman *et al.*, 2008). GFP-based fluorescent proteins are inherently fluorescent; in contrast, iLOV fluorescence relies on its chromophore co-factor, flavin mononucleotide (FMN). iLOV expression in goat epithelium cells was confirmed by confocal microscopy, flow cytometry and live-cell imaging microscopy, showing that dependency on the cellular co-factor FMN did not impede detection of its fluorescence. Furthermore, we did not observe an improvement in fluorescence as judged by live-cell imaging on addition of riboflavin to the media of cells infected with iLOV-FMDV (data not shown). iLOV may exhibit a number of advantages over GFP-based fluorescent proteins. Techniques such as live-cell imaging utilize recurrent laser scanning, which exposes fluorescent marker proteins to high light intensities. This can cause irreversible bleaching of GFP; however, iLOV has been shown to have a latent photochemistry that recovers spontaneously. In agreement, we observed no obvious reduction of fluorescence of iLOV in our live-cell imaging experiments. The GFP fluorophore can take a period of hours to mature, hindering real-time studies investigating the early events of viral life cycles (Cubitt *et al.*, 1995; Wright *et al.*, 2007). Following the direct addition of virus to cell media, we commonly observed iLOV expression after 2 h. Taking into account the time required for the virus to enter the cell, uncoat and produce positive-strand templates, these results show that the initial detection of iLOV coincides with that of the non-structural proteins (García-Briones *et al.*, 2006) and confirm that iLOV is a feasible alternative for investigating early viral events.

The smaller size of iLOV compared to GFP may also alleviate problems associated with dysfunctional GFP fusions. In this study, iLOV was expressed as an N-terminal fusion of FMDV 2A. Although FMDV 2A is only 18 amino acids long it may have an additional, as yet unreported, role other than being involved in processing the viral polyprotein. The non-structural proteins of RNA viruses are often pleiotropic, having multifunctional roles to compensate for the small coding capacity of their genomes. iLOV-FMDV exhibited a similar rate of replication and plaque morphology to the parental virus, suggesting that if the 2A product has additional functions these were not impeded. In addition, we have constructed a variant infectious rFMDV (termed iLOV-3C-FMDV) in which a 3C cleavage site was introduced at the C terminus of iLOV in order to generate separate iLOV and 2A products. The spatial and temporal expression of iLOV protein was comparable, as judged by live-cell imaging, in goat epithelium cells infected with iLOV-FMDV or iLOV-3C-FMDV (data not shown).

Although iLOV fluorescence was recoverable in comparison to other GFP constructs or fluorophores routinely used by our group, the fluorescent signal for iLOV appeared relatively faint. As discussed above, quenching of iLOV fluorescence as a result of our cloning strategy is unlikely, as expression of iLOV as a non-fusion product gave similar results. Other factors, including the accumulation and turnover of 2A in the cytoplasm or simply its inherent fluorescent properties, may play a role. Numerous GFP mutants have been engineered to optimize its use as a molecular tool for research. These mutations improved the spectral characteristics of GFP, changed the colour of the emitted fluorescence and prevented dimerization (Shaner *et al.*, 2005). Therefore, like GFP, iLOV may benefit from further optimization to enhance its spectral characteristics when expressed in mammalian cells.

A number of recent studies have used replication-competent viruses that express easily traceable reporter genes to study the dynamics of viral infections. Manicassamy *et al.* used a recombinant GFP-expressing influenza virus to characterize viral dissemination in mice and identified a diverse set of target host cells, including macrophages, monocytes, neutrophils, respiratory dendritic cells, B, NK, CD4 and CD8 cells; in addition, the GFP influenza virus was used to investigate the action of antiviral agents on these cell types (Manicassamy *et al.*, 2010). Sánchez-Puig and co-workers infected fresh preparations of human peripheral blood leucocytes with vaccinia virus recombinants expressing GFP and showed a strong bias towards infection of monocytes, B lymphocytes and NK cells (Sánchez-Puig *et al.*, 2004). iLOV-FMDV offers a comparable tool for the characterization of the FMDV life cycle. Its fluorescence and stability properties make it suitable for *in vitro* live-cell experiments investigating FMDV replication in cultured cells, *in vivo* studies using the mouse as a model system to characterize FMDV infection and *ex* *vivo* analysis of infected livestock tissues to identify rare host cell types that facilitate viral replication (Fernández *et al.*, 1986; Reid *et al.*, 2011; Salguero *et al.*, 2005). We have recently shown plasmacytoid dendritic cells (pDC) isolated from bovine mesenteric lymph nodes, pseudo-afferent lymph and blood produce type I IFN in response to immune-complexed FMDV. Future studies will utilize iLOV-FMDV to investigate dissemination of FMDV in mice, as well as the infection status of pDCs *in vivo*.

## Methods

### 

#### Construction of viruses.

Recombinant copy viruses, containing either full-length *Renilla* luciferase ORF, full-length GFP ORF or increasing portions of the GFP ORF (clones T2, T3, T4, T5, T6 or T7), were constructed using reverse genetics and existing clones. Briefly, an FMDV O1K/O UKG35 chimeric clone encoding the VP2, VP3, VP1 and 2A products of FMDV UKG/35/2001 and the Lpro, VP4, 2B, 2C, 3A, 3B, 3C and 3D products of FMDV O1K/O1Manisa was used as the parental construct for all cloning in this study. This recombinant copy virus has been previously described (Bøtner *et al.*, 2011; Seago *et al.*, 2012). To facilitate cloning, a unique *Avr*II restriction site was inserted into the genome of the O1K/O UKG35 chimera between DNA encoding the VP1 and 2A products. This was done by performing two consecutive rounds of PCR amplification using the QuikChange Lightning Mutagenesis kit (Agilent Technologies) according to the manufacturer’s instructions. Respective GFP and *Renilla* luciferase inserts were PCR amplified from existing clones and ligated into the *Avr*II restriction site. The primers used for amplification of the *Renilla* luciferase and GFP DNA inserts are listed in Table S1 (available in the online version of this journal).

#### Preparation of infectious RNA, electroporation and transfection.

RNA was transcribed from the infectious clones using the MEGAscript T7 kit (Invitrogen). The infectious RNA was first electroporated into BHK-21 cells using a Bio-Rad Gene PulsarTM (two pulses at 0.75 kV and 25 μF). After 24 h, the cells were thawed in their growth media and clarified by centrifugation, the supernatant of which contained the initial virus stock (termed ‘passage 0’, P0). A goat epithelium cell line expressing the principal FMDV receptor (integrin αvβ6) was subsequently used to passage the tagged viruses (P1) (Brehm *et al.*, 2009). Cells were infected for 24 h between passages. Electroporation of RNA was performed using the TransIT mRNA Transfection kit (MoBiTec) according to the manufacturer’s instructions.

#### Genome amplification and sequencing.

Total RNA was extracted using TRIzol reagent (Invitrogen) and the respective region of the viral RNA genome was reverse transcribed and amplified by PCR using a One-Step RT-PCR kit (Qiagen). Sequencing reactions were then performed using an aliquot of the purified PCR product and the BIG Dye Terminator v3.1 cycle sequencing kit (Applied Biosystems).

#### Western blot analysis.

For Western blots, proteins were separated by SDS-PAGE (12 % acrylamide) and then transferred to nitrocellulose membranes (Hybond-C Extra, Amersham Biosciences). Membranes were blocked with dried skimmed milk in PBS containing 0.1 % Tween 20. Primary monoclonal antibodies used were: 2C2 (mouse anti-FMDV 3A) (De Diego *et al.*, 1997), mouse anti-γ-tubulin (Sigma-Aldrich), rabbit anti-GFP (Abcam) and rabbit anti-iLOV (Chapman *et al.*, 2008). Bound primary antibodies were detected by horseradish peroxidase-conjugated anti-mouse or anti-rabbit antibodies (Bio-Rad).

#### Immunofluorescence microscopy.

Baby hamster kidney-21 cells (BHK-21) cultured on glass coverslips were transfected with GFP-FMDV transcripts using the TransIT mRNA transfection kit (Cambridge Bioscience) according to the manufacturer’s instructions. Goat epithelium cells (ZZ-R127) (Brehm *et al.*, 2009) cultured on glass coverslips were incubated with iLOV-FMDV. Uninfected cells were included as a control. After 5 h, cells were fixed, permeabilized, washed and blocked (0.5 % BSA in PBS). FMDV non-structural protein 3A was labelled using monoclonal antibody 2C2 (De Diego *et al.*, 1997). Goat anti-mouse Molecular Probes Alexa-Fluor-conjugated secondary antibodies (Invitrogen) were used, and nuclei were stained with DAPI (Sigma-Aldrich). All data were sequentially collected using a Leica SP2 scanning laser confocal microscope.

#### Plaque assay.

Confluent monolayers of goat epithelium cells were infected with serial dilutions of FMDV O1K/O UKG35 virus stocks, overlaid with indubiose and incubated for 24 to 48 h at 37 °C. The cells were then fixed and stained (4 % formaldehyde in PBS containing methylene blue) before removal of the overlay (Jackson *et al.*, 2000).

#### Flow cytometry.

Goat epithelium cells were either mock infected or infected with iLOV-FMDV or parental-FMDV at an m.o.i. of 2. After 4 h at 37 °C, the cells were fixed with 4 % paraformaldehyde, washed in PBS and permeabilized in FACS diluent (0.2 % saponin/1 % BSA/autoMACS Running buffer solution, Miltenyi Biotec, UK). All subsequent steps were carried out in FACS diluent containing 5 % normal goat serum (Sigma-Aldrich). Cells were blocked for 20 min at room temperature then labelled with either anti-FMDV non-structural protein 3A monoclonal antibody 2C2 (De Diego *et al.*, 1997), anti-FMDV capsid monoclonal antibody IB11 (IgG2a) (Juleff *et al.*, 2008) or isotype control monoclonal antibodies TRT1 (IgG1) and TRT3 (IgG2a) (Cook *et al.*, 1993). Antibodies were directly conjugated with Zenon Alexa 647 fluorophores (Invitrogen). After incubation for 30 min at room temperature, the cells were washed and resuspended in PBS. Using a MACSQuant Analyser (Miltenyi Biotec), single cell populations were gated based upon forward and side scatter characteristics. The specific fluorescence of iLOV and Alexa-Fluor 647 was measured upon excitation at 488 nm and 635 nm, respectively. Data from 10 000 events were recorded, and the data was analysed using FSC Express version 3 (De Nova Software, USA).

#### Live-cell imaging.

Goat epithelium cells were grown on Lab-Tek chambered coverglass slides (Nunc) and either mock infected or infected with iLOV-FMDV or parental-FMDV at an m.o.i. of 2. After 2 h, cell culture medium was removed and replaced with Leibovitz’s L-15 medium (Gibco). Images were acquired with a Leica SP2 confocal laser microscope with DMIRE2 inverted microscope stand in a microscope chamber kept at 37 °C. The cells were viewed by differential interference contrast optics, iLOV was excited at 488 nm, and the emission fluorescence levels were detected by using a photo-multiplicator between 500 and 570 nm. Time-lapse imaging of iLOV-FMDV-infected cells was performed by acquiring images every 20 min over 5 h.
